# Single-cell microbiota phenotyping reveals distinct disease and therapy-associated signatures in Crohn’s disease

**DOI:** 10.1080/19490976.2025.2452250

**Published:** 2025-01-15

**Authors:** Lisa Budzinski, Gi-Ung Kang, René Riedel, Toni Sempert, Leonie Lietz, René Maier, Janine Büttner, Bettina Bochow, Marcell T. Tordai, Aayushi Shah, Amro Abbas, Tanisha Momtaz, Jannike L. Krause, Robin Kempkens, Katrin Lehman, Gitta A. Heinz, Anne E. Benken, Stefanie Bartsch, Kathleen Necke, Ute Hoffmann, Mir-Farzin Mashreghi, Robert Biesen, Tilmann Kallinich, Tobias Alexander, Bosse Jessen, Carl Weidinger, Britta Siegmund, Andreas Radbruch, Anja Schirbel, Benjamin Moser, Hyun-Dong Chang

**Affiliations:** a German Rheumatology Research Centre Berlin – A Leibniz Institute, Berlin, Germany; b Department for Cytometry, Institute of Biotechnology, Technische Universität Berlin, Berlin, Germany; c Bioinformatics and Computational Biology, Department of Cardiology, University Medical Centre Hamburg-Eppendorf, Hamburg, Germany; d Department of Hepatology and Gastroenterology, Campus Charité Mitte, Charité, Universitätsmedizin Berlin corporate member of Freie Universität Berlin and Humboldt-Universität zu Berlin, Berlin, Germany; e Department of Gastroenterology, Infectious Diseases and Rheumatology, Campus Benjamin Franklin, Charité – Universitätsmedizin Berlin corporate member of Freie Universität Berlin and Humboldt-Universität zu Berlin, Berlin, Germany; f School of Pharmacy, BRAC University, Dhaka, Bangladesh; g Department of Rheumatology, Campus Charité Mitte, Charité – Universitätsmedizin Berlin corporate member of Freie Universität Berlin and Humboldt-Universität zu Berlin, Berlin, Germany; h Department of Paediatric Respiratory Medicine, Immunology and Critical Care Medicine, Charité Campus Virchow, Charité Universitätsmedizin Berlin, Berlin, Germany; i BIH Charité Clinician Scientist Program; j DRK Kliniken Berlin, Clinic for internal medicine – Gastroenterology, Haematology and Oncology, Nephrology, Centre for chronic gastrointestinal inflammations, Berlin, Germany

**Keywords:** Single-cell analysis, microbiota flow cytometry, microbiota phenotyping

## Abstract

IgA-coated fractions of the intestinal microbiota of Crohn’s disease (CD) patients have been shown to contain taxa that hallmark the compositional dysbiosis in CD microbiomes. However, the correlation between other cellular properties of intestinal bacteria and disease has not been explored further, especially for features that are not directly driven by the host immune-system, e.g. the expression of surface sugars by bacteria. By sorting and sequencing IgA-coated and lectin-stained fractions from CD patients microbiota and healthy controls, we found that lectin-stained bacteria were distinct from IgA-coated bacteria, but still displayed specific differences between CD and healthy controls. To exploit the discriminatory potential of both, immunoglobulin coated bacteria and the altered surface sugar expression of bacteria in CD, we developed a multiplexed single cell-based analysis approach for intestinal microbiota. By multi-parameter microbiota flow cytometry (mMFC) we characterized the intestinal microbiota of 55 CD patients and 44 healthy controls for 11-parameters in total, comprising host-immunoglobulin coating and the presence of distinct surface sugar moieties. The data were analyzed by machine-learning to assess disease-specific marker patterns in the microbiota phenotype. mMFC captured detailed characteristics of CD microbiota and identified patterns to classify CD patients. In addition, we identified phenotypic signatures in the CD microbiota which not only reflected remission after 6 weeks of anti-TNF treatment, but were also able to predict remission before the start of an adalimumab treatment course in a pilot study. We here present the proof-of-concept demonstrating that multi-parameter single-cell bacterial phenotyping by mMFC could be a novel tool with high translational potential to expand current microbiome investigations by phenotyping of bacteria to identify disease- and therapy-associated cellular alterations and to reveal novel target properties of bacteria for functional assays and therapeutic approaches.

## Introduction

The human intestinal microbiota are intricately linked to human health. They play an essential role in host energy homeostasis and metabolism, but also contribute significantly to the maturation of the immune system as a steady interaction partner. In consequence, various human diseases, ranging from metabolic to chronic inflammatory diseases and cancer to neurological disorders,^1^ have been associated with alterations in the composition of the intestinal microbiota. Typically, the microbiota is characterized by sequencing the highly conserved 16S rRNA gene, encoding a bacteria-specific ribosomal RNA of the small ribosomal subunit, which contains variable regions allowing the determination of phylogenetic relationships between bacteria, and thus taxonomic classification. Applied to cohorts of, e.g., patients and healthy controls, 16S rRNA sequencing has revealed alterations in the abundance or presence of certain bacterial taxa, the overall compositional diversity, and the microbial load in multiple diseases. However, the vast inter-individual diversity in the microbiota composition in humans has interfered with the identification of disease-specific taxonomic microbial signatures as biomarkers. Although many studies have contributed to research on the pathogenic potential of certain intestinal microbes, the lack of ubiquitousness and robustness have so far precluded routine clinical application toward the benefit of patients.^2–4^

Results from the Human Microbiome Project have indicated that despite high taxonomic diversity between individuals, functions of the microbial community are rather conserved, suggesting that the microbial composition is governed by functionality and interaction with the host.^5^ In patients with Crohn’s disease (CD), the microbiota have been found to possess distinct metabolomic profiles compared to healthy donors.^6^ Additionally, drastic changes in the host-microbiota interaction and a modified immune response toward components of the microbiota are reflected by an altered immunoglobulin secretion and coating of intestinal bacteria with host immunoglobulins in CD.^7–10^ Consequently, it appears promising to investigate the microbiota as a community of single cells, each a functional unit, shaped by their micro-environment and host-derived factors. Alongside host immunoglobulins, surface sugar moieties appear promising to reflect on microbiota-host interactions as surface glycosylation of bacteria may correlate with metabolic activity, nutritional state and an inflammatory microenvironment resulting in altered interplay between bacteria or with the host.^11^ Clinical relevance of lectin binding to bacteria in CD has been suggested by linking the binding of the host lectin intelectin-1 to the pathogenesis of intestinal inflammation.^12^ However, alterations regarding sugar moieties of the intestinal microbiota in CD have not yet been investigated.

Flow cytometry is a widely used tool to rapidly investigate cellular properties on single-cell level, but its potential has not yet been fully explored for microbiota analyses.^13^ Microbiota flow cytometry (MFC) has been shown to be an effective method to capture compositional dynamics of microbial communities,^14–17^ assessing their complexity and compositional changes by light scatter properties and quantitative DNA staining.^14,15,18^ We and others have previously demonstrated the utility of MFC for monitoring microbiota dynamics longitudinally, both *in vitro* and *ex vivo* in murine colitis,^16,19^ during chemotherapy-treatment of patients with hematological malignancy^17^ and to discriminate CD patients from healthy donors.^20^

Here, we present an advanced multi-parameter microbiota flow cytometry (mMFC) approach to analyze single bacterial cells in complex communities of the human intestinal microbiome phenotypically. Initially, by IgA-Seq, we confirmed that IgA-coating reflects adapted immune responses to shifts in the microbial community as the IgA-coated fractions in CD patients were mainly composed of taxa that also characterized the CD dysbiosis obtained by 16S rRNA sequencing. In contrast, lectin-Seq, i.e. 16S rRNA-based identification of bacteria based on staining and sorting with plant-derived lectins, for wheat germ agglutinin (WGA, target: N-acetyl-glucosamine) and peanut agglutinin (PNA, target: galactose), indicated that the surface sugar expression may mainly be a dynamic property of the microbes and sensitive to the micro-environment.

We have combined the isotype-specific detection of coating with host immunoglobulins (IgA1, IgA2, IgM and IgG) with the staining of sugar residues by lectins on the bacterial surface on a set of stool samples of CD patients to interrogate their microbial phenotypes for disease- and therapy-related biosignatures.^21^ Comparable to what has been described for blood cell phenotyping, we have reduced the complexity of high-dimensional data by clustering microbial cells by phenotypic similarity. Using machine-learning, we could classify CD patients despite cohort heterogeneity from healthy controls. In addition, microbiota phenotyping revealed a biosignature in CD patients stratifying patients achieving remission criteria in response to anti-TNF therapy during but also prior to start of the therapy.

In summary, we demonstrate that mMFC-based single-cell phenotyping of intestinal bacteria is a robust approach to assess altered microbiota properties reflecting the adaption of the mucosal immune response to taxonomic shifts and phenotypic features of a dysbiotic microbial community in an inflammatory environment so far overlooked by taxonomic profiling.

## Results

### Host IgA-coating but not lectin-staining mark known colitogenic bacteria

We isolated bacteria based on IgA-coating, WGA-binding and PNA-binding, respectively, from 14 CD patients (CD cohort 1) and 8 healthy controls with fluorescence-activated cell sorting and subjected them to full-length 16S rRNA gene sequencing to characterize their composition in more detail. We used beta-dissimilarity by Bray-Curtis to compare the composition between samples or sorted fractions, which considers presence and distribution of taxa and ranges from 0 for samples of equal composition to 1 for maximally different samples. To visualize differences between the samples of the two groups, we computed the distance in beta-dissimilarity between donors and projected the distribution to a principal coordinate analysis plot.

**Figure 1.**
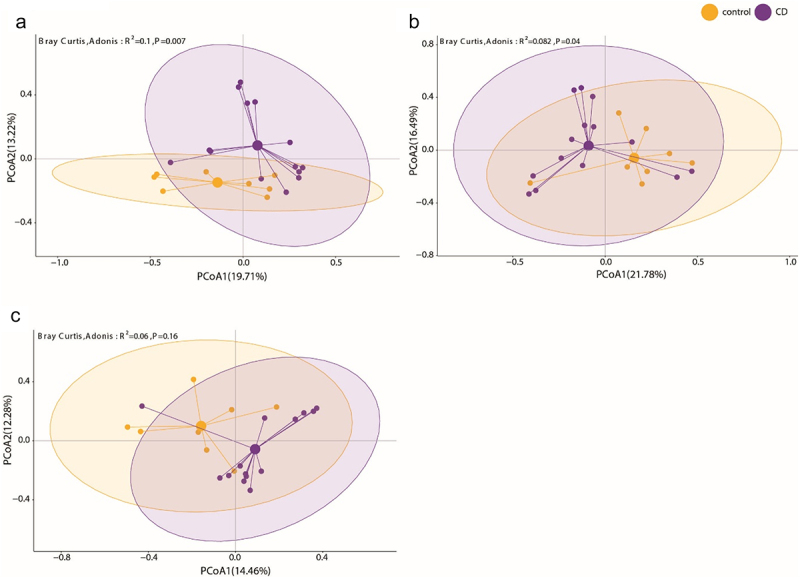
IgA-seq and lectin-seq potentially reveal functionally distinct bacterial taxa. Bacteria from CD patients (*n* = 14, purple) and healthy donors (*n* = 8, orange) were isolated by fluorescence activated cell sorting (FACS) according to (a) IgA-coating, (b) staining with wheat germ agglutinin (WGA) and (c) staining with peanut agglutinin (PNA) and submitted to full-length 16S rRNA gene sequencing. (a, b, c) the principal coordinate projection represent the Bray-Curtis dissimilarity of the taxonomic composition of the respective sorted fractions of CD and healthy donor samples.

The taxonomic composition of the IgA-coated fractions of both cohorts was significantly different between CD and healthy samples (Figure 1a, R^2^ = 0.1, *p* = 0.007). Bacterial taxa found to be enriched in the IgA-coated fraction in CD patients included *Ruminococcus gnavus sp*. and *Escherichia-Shigella sp*. which were also dominant in CD samples on bulk level (Supplementary Figure S1, Supplementary Figure S2). While in healthy donors, *Faecalibaterium sp., Subdoligranulum sp*. and *Lachnospiraceae NK4A136 sp*. were enriched in the IgA-coated bacteria and were also dominant in the bulk sample. A less apparent but still significant separation was observed between CD and healthy controls when considering WGA-stained bacteria (Figure 1b, R^2^ = 0.082, *p* = 0.04). We here found *Ruminoccous gnavus group sp. and Escherichia-Shigella sp*. enriched exclusively in the CD sorts, while again *Feacalibacterium prausnitzii* and *Lachnospiraceae NK4A136 sp*. were representatives for the sorted fractions from controls (Supplementary Figure S3A). Similar results were found when sorting the PNA-positive bacteria, albeit they did not show significant differences in composition between CD and healthy (Figure 1c, R^2^ = 0.06, *p* = 0.16). Here, *Ruminoccous gnavus group sp*. was not enriched in CD. *Escherichia-Shigella sp*. and *Lachnoclostridium sp*. were still found enriched among the PNA-stained bacteria in CD (Supplementary Figure S3B). Overall, *Veillonella sp*. which was prominently found in CD samples when looking at the total microbiome, was not found in in any of the sorts. Instead *Blautia sp*. and *Stenotrophomonas sp*. which were not conspicuous in the total microbiome were found significantly enriched in the respective enriched populations. This raised the question, how well the taxonomic composition and microbial phenotypes represent the CD and healthy state.

### Single-cell analysis reveals Crohn’s disease specific phenotypic alterations of the microbiota

**Figure 2.**
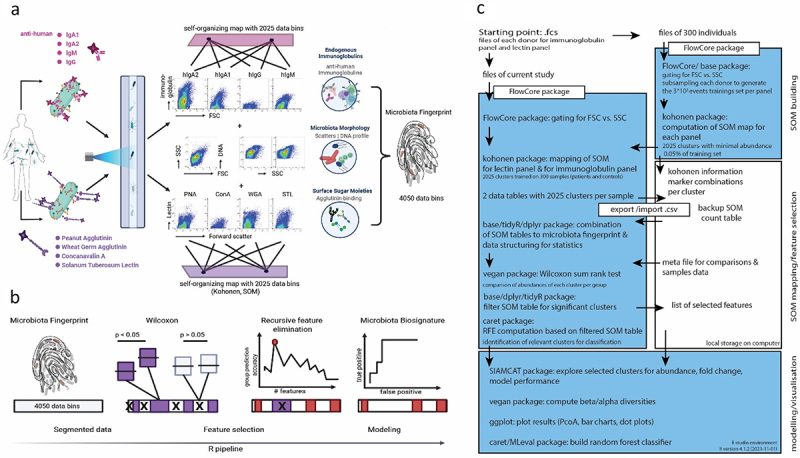
Microbiota phenotyping by flow cytometry and machine learning to identify specific biosignatures in human stool samples. (a) Human intestinal bacteria from stool samples were stained with monoclonal antibodies specific for the human immunoglobulins IgA1, IgA2, IgM and IgG and with the lectins peanut agglutinin (PNA), wheat germ agglutinin (WGA), *Solanum tuberosum* lectin (STL) and concanavalin a (ConA). Each staining panel also included the cell wall/membrane-permeable DNA dye hoechst 33,342. After data acquisition in a flow cytometer, the cells of each staining panel were clustered according to a previously defined self-organizing map (SOM) into 2025 clusters. The abundance of cells per cluster in the total of 4050 clusters represented the overall microbiota phenotype of a sample. (b) The clusters were filtered by Wilcoxon statistical evaluation and recursive feature elimination to select the significant and most relevant clusters defining the specific microbiota biosignature for random forest model-based classification of disease and comparison of samples by Bray-Curtis dissimilarity (β-diversity) projection. (c) Outline of all relevant data processing steps and used packages (R) for computing a microbiota phenotype.

To address this in more detail, we enlarged our cohort and characterized 55 CD samples (CD cohort 1) and compared their microbiota phenotype to healthy controls (HC, *n* = 44). Bacteria were stained with the DNA dye Hoechst 33,422 and isotype-specific anti-human IgA1, IgA2, IgM and IgG antibodies to assess surface coating in one staining panel and with the lectins wheat germ agglutinin (WGA, target: N-acetyl-glucosamine), peanut agglutinin (PNA, target: galactose), *Solanum tuberosum* agglutinin (STL, target: N-acetyl-glucosamine) and Concanavalin A (ConA, target: mannose) in a second panel to assess surface sugar expression (Figure 2a).^21^ The selection of lectins was based on commercial availability, diversity of their staining patterns to represent the variety of intestinal microbiota phenotypes, and recognition of sugars which have the potential to interact with lectins of the immune system, e.g. galactose and mannose.^22–24^ To analyze the multi-dimensional data of each donor, we applied a dimensionality-reducing clustering algorithm to group cells with similar phenotypic properties. For clustering, we applied a map (SOM) with 2025 clusters, which was trained on a larger data set (see methods), to the data of each staining panel. We then combined the clusters of both panels to a total of 4050 clusters to describe the microbiota phenotype of each sample. The frequency of cells per cluster was used to quantify the overall difference (Bray-Curtis dissimilarity) between samples and used for the determination of specific biosignatures (Figure 2). Taking all clusters into account, we observed a significant difference between CD and healthy controls, however, the high interindividual variability between all the samples resulted in a notable overlap between the cohorts (Supplementary Figure S4A, R^2^ = 0.05, *p* = 0.001).

**Figure 3.**
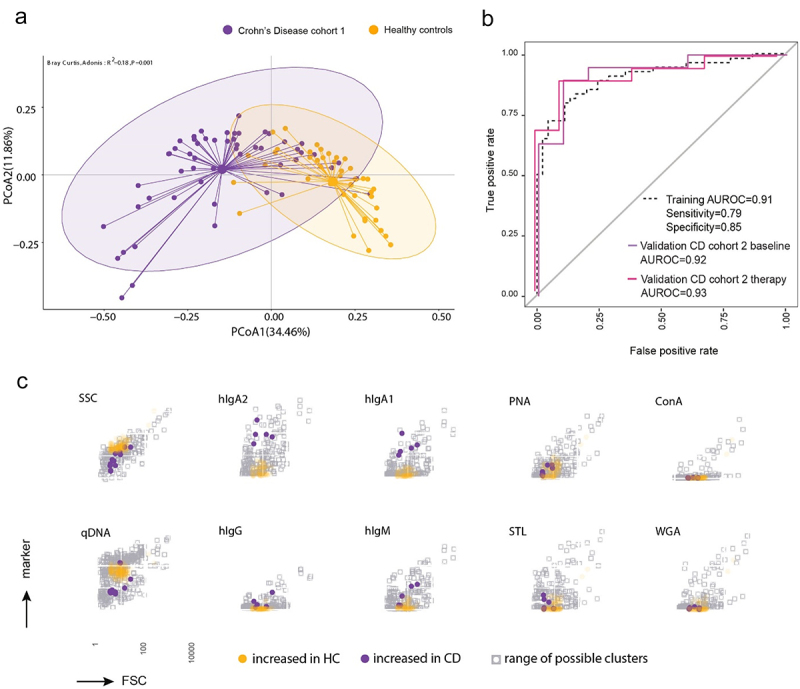
Microbiota phenotyping reveals a specific cytometric biosignatures of Crohn’s disease. Samples of CD patients from cohort 1 (*n* = 55) and healthy controls (*n* = 44) were stained for host immunoglobulins and surface sugars. Following SOM clustering and cluster selection, 187 clusters were identified. (a) Principal coordinate projection representing the Bray-Curtis dissimilarity between samples of CD cohort 1 and healthy controls according to the 187 selected clusters. (b) The 187 clusters were used to train a random forest model classifying between CD patients and healthy controls. The model was validated with 19 patients from an independent CD cohort, sampled at two time points: before therapy (baseline) and after 6 weeks of anti-tnf therapy (therapy) and 10 new healthy donors. The performance of the binary classifier model is illustrated by the AUROC curves. (c) Projection of the selected clusters containing increased abundance of bacterial cells in either CD patients or healthy controls visualizing the mean fluorescence intensity in each stained parameter in relation to all clusters.

By statistical testing (Wilcoxon) and recursive feature elimination (RFE) (Figure 2b,c), 187 out of the 4050 clusters were selected that would best represent a CD-specific phenotypic microbiota biosignature in comparison to healthy controls. This selection resulted in an improved separation of the CD from the healthy samples (PCoA1: 34.46%, PCoA2: 11.86%, R^2^ = 0.18, *p* = 0.001, Figure 3a). Many of the 187 clusters discriminating CD vs. healthy showed a decreased abundance in the CD samples (Supplementary Figure S5, S6) and were characterized by a higher DNA and SSC signal (Figure 3c). Clusters enriched in CD samples contained primarily microbial cells with a low DNA signal and increased coating with hIgA1/2 (Figure 3c, Supplementary Figure S5). The lectin staining was not obviously increased in CD patients (Figure 3c, Supplementary Figure S6).

We trained a random-forest model-based classifier based on these 187 clusters on CD cohort 1, which discriminated CD samples from healthy controls with an AUROC = 0.91 (specificity = 0.85 and sensitivity = 0.79) (Figure 3b). To validate the classification and thus the biosignature, we analyzed a second independent cohort of 19 CD patients, selected directly the 187 previously identified clusters and tested the performance of our classification model on this sample set. The 19 patients were each sampled twice, first prior (CD cohort 2 baseline, active CD) and second 6 weeks in anti-TNF therapy (CD cohort 2 therapy). Our classification model was also able to classify CD cohort 2 despite heterogeneity in disease activity with an AUROC = 0.92 for the baseline and 0.93 for 6w therapy samples, respectively (Figure 3b).

**Figure 4.**
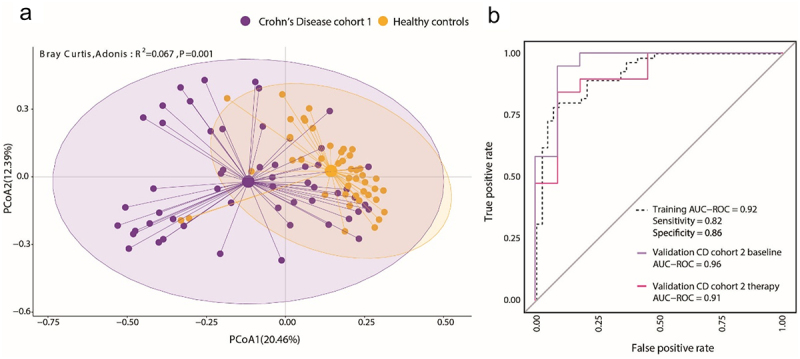
The microbiome composition is altered in Crohn’s disease patients. Samples of CD patients from cohort 1 (*n* = 55) and healthy controls (*n* = 44) were analyzed by 16S rRNA V3/V4 amplicon sequencing. Identified genus level taxa also underwent selection as described resulting in 82 selected taxa. (a) Principal coordinate projection representing the Bray-Curtis dissimilarity between samples of CD cohort 1 and healthy controls according to the 82 selected taxa. (b) A random forest model classifier to distinguish between CD patients and healthy controls was trained with the 82 taxa. The model was validated with the 19 patients from CD cohort 2, sampled at two time points: before therapy (baseline) and after 6 weeks of anti-tnf therapy (therapy) and with the 10 new healthy donors. The performance of the taxonomy-based binary classifier model is illustrated by the AUROC curves.

In parallel, all samples were analyzed by 16S rRNA gene amplicon sequencing to determine the composition of the samples on taxonomic level. The identified taxa were also further filtered by Wilcoxon statistical test and RFE. In total, 82 taxa were selected for the best discrimination between CD patients and healthy controls (Supplementary Figure S2). The dissimilarity between CD and healthy controls based on these 82 taxa increased to R^2^ = 0.067 (*p* = 0.001) (Figure 4a) compared to R^2^ = 0.047 (*p* = 0.001) when all taxa were considered (Supplementary Figure S4B). The discrimination between healthy controls and CD samples based on these 82 taxa with a random-forest model performed similarly to the cytometric profiling in training (AUROC = 0.92, specificity = 0.86, sensitivity = 0.82) and in validation with the second CD cohort (AUROC = 0.96 for baseline and 0.91 6w therapy samples) (Figure 4b). The main discriminator was an increased abundance of *Ruminoccocus gnavus sp*. in CD patients.

Thus, our results demonstrate that microbiota phenotyping was able to robustly classify CD patients similarly to 16S rRNA sequencing.

### Identification of a microbiota signature reflecting therapy response

**Figure 5.**
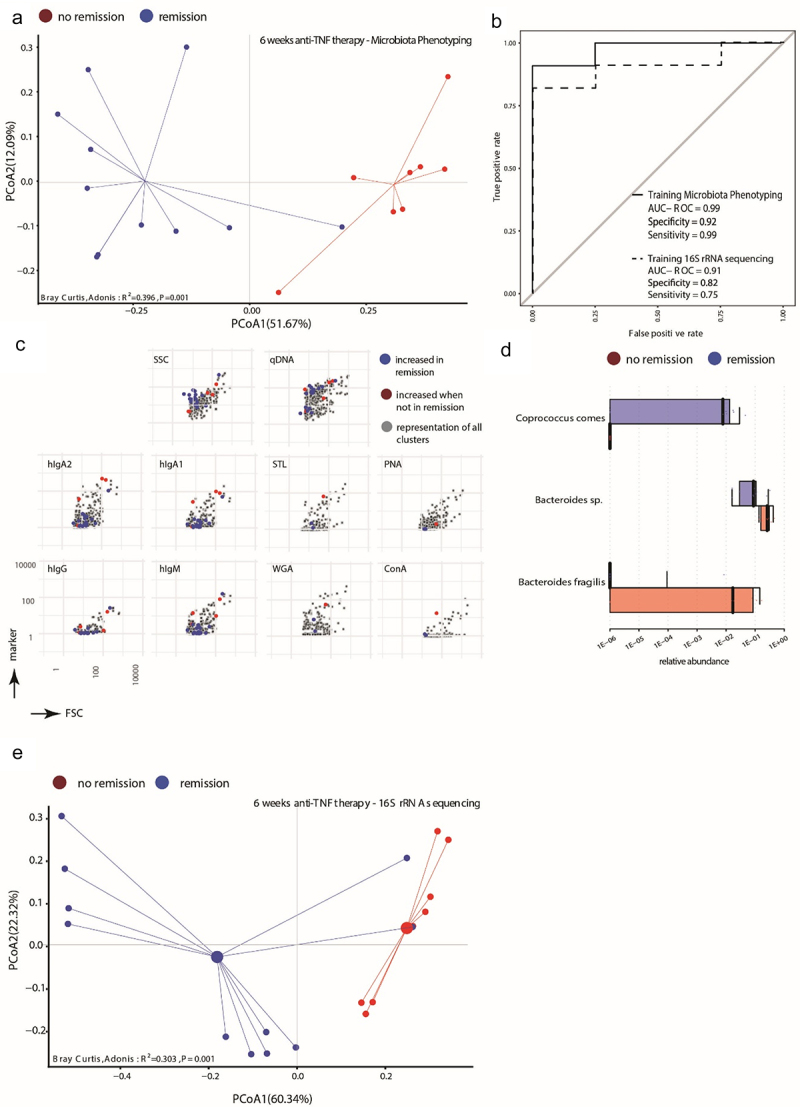
Phenotypic properties of the microbiota and several taxa correlate with therapy success for Crohn’s disease patients after 6 weeks of anti-tnf therapy. 19 CD patients (CD cohort 2) were sampled before initiation of treatment with adalimumab and 6 weeks later, at which timepoint the patients were stratified into those achieving remission (Harvey-Bradshaw-index, HBI < 5, *n* = 11) and those not achieving remission criteria (HBI ≥5, *n* = 8) (a) Principal coordinate projection representing the Bray-Curtis dissimilarity between samples of remission (blue) and no remission (red) according to 24 selected cluster 6 weeks after initiation of adalimumab treatment. (b) A random forest model classifier to distinguish between CD patients in remission and patients not in remission after 6 weeks of adalimumab treatment was trained with the 24 selected phenotypic clusters (solid line) or with 3 taxa (dotted line) identified by 16S rRNA amplicon sequencing. The performance of the respective classifier model is illustrated by the AUROC curves. (c) Projection of the selected clusters containing increased abundance of bacterial cells in CD patients in remission or not in remission visualizing the mean fluorescence intensity in each stained parameter in relation to all clusters. (d) Abundance of the bacterial taxa significantly differentially abundant between patients in remission or not in remission following 6 weeks of adalimumab treatment. Indicated are the median abundance, 95% confidence interval and coefficient of variation. (e) Principal coordinate projection representing the Bray-Curtis dissimilarity between samples of remission (blue) and no remission (red) according to the 3 taxa 6 weeks after initiation of adalimumab treatment.

We next set out to test, whether the response of CD patients to the treatment with TNF blockers is reflected in the microbial signature. For this we analyzed the samples of CD cohort 2. The 19 CD patients of cohort 2 had received anti-TNF therapy (adalimumab) either for the first time (*n* = 12) or after an anti-TNF therapy break of at least 4 weeks (*n* = 7, details in Table 1) and were profiled with mMFC and 16S rRNA gene sequencing at baseline prior to anti-TNF therapy and 6 weeks after therapy initiation. At 6 weeks, the CD patients were grouped into those having achieved remission or not according to the Harvey-Bradshaw index (HBI), which assesses, e.g. the general well-being of the patient, their stool frequency and abdominal mass.^25^ 11 out of the 19 patients fulfilled remission criteria (HBI <5) while 8 patients did not (HBI ≥5). At baseline, the remission group exhibited lower, but not significantly different, disease score values for HBI (remission: mean HBI = 11.4 ± 4.4, no-remission: mean HBI = 12.4 ± 3.8) and Crohn’s disease activity index (CDAI) (remission: mean CDAI = 305, no-remission: mean CDAI = 308). At the initiation of therapy both groups showed elevated CRP levels (remission: 34, no-remission: 26). The ultrasonography-based Limberg score^26^ was similar in both groups (Table 2). In patients of the remission group the HBI score was reduced to 3.2 ± 3.2 (*p* = 0.04), while in patients attributed to the no-remission group the HBI score was reduced to 5 ± 2.9 (*p* = 0.014) indicating that the majority of patients responded to the therapy even when not achieving the remission criteria of the study.^27^ When sampling after 6 weeks of therapy, we detected significant differences both in the phenotypic biosignature and in the taxonomic composition of the intestinal microbiota between remission and no-remission patients (Figure 5a,e). 24 phenotypic clusters were identified to discriminate microbiota of remission patients and no-remission patients resulting in a clear separation of the two groups by Bray-Curtis dissimilarity (R^2^ = 0.396, *p* = 0.001) (Figure 5a). Accordingly, the random-forest model classified the samples into remission and no-remission with an AUROC = 0.99 (specificity = 0.92, sensitivity = 0.99) (Figure 5b, solid line). The clusters dominant in no-remission patients comprised bacteria displaying increased coating with the host immunoglobulins hIgA1/2 and hIgM compared to remission patients (Figure 5c, Supplementary Figure S7A). This signature associated with remission was not present at baseline, as the 24 clusters did not significantly differentiate between the groups (*p* = 0.472, Supplementary Figure S7B) at this sampling time point. On the taxonomic level, only 3 taxa were identified. *Coprococcus comes* was strongly associated with remission, with the limitation that it was only detectable in six out of 11 remission patients. *Bacteroides fragilis* was associated with no remission, which was detectable in only 5 out of the 8 no-remission patients (Figure 5d). Nevertheless, the Bray-Curtis dissimilarity projection based on the three selected taxa showed a clear separation between remission and no-remission (R^2^ = 0.303, *p* = 0.001, Figure 5e). The random-forest model for the 16S rRNA sequencing data performed with an AUROC = 0.91 (specificity = 0.82, sensitivity = 0.75) (Figure 5b, dotted line). Combining phenotypic signature with 16S sequencing did not improve differentiation of the remission and no-remission group at week 6 (Supplementary Figure S8A), as in the combined RFE, only phenotypic clusters and no taxa were selected (data not shown). Finally, we tested whether the therapy-induced changes in the phenotypic signature of the microbiota of CD patients achieving remission resulted in a convergence toward the microbial biosignature of healthy controls. For this, we applied the selected 187 clusters of the CD *vs*. healthy controls biosignature (Figure 3) to the samples of CD cohort 2. We then compared the change in dissimilarity of each patient from baseline to week 6 to the mean value of all healthy controls (Supplementary Figure S9A). Patients that achieved remission showed a reduced dissimilarity to healthy controls, albeit not reaching statistical significance, compared to the no remission patients. This trend is also represented in the smaller average distance of the remission samples to the healthy cohort in the principal coordinate projection (Supplementary Figure S9B). In summary, the success of anti-TNF therapy in CD patients is reflected both in a specific alteration in the phenotypic signature and to some extent also in the taxonomic composition of the intestinal microbiota.

**Table 1.** Donor information.

	CD cohort 1	CD cohort 2 (remission group)		CD cohort 2 (no-remission group)		controls
female/male	29/25	3/8		4/4		35/20
age	43 ± 13	36 ± 10		43 ± 10		48 ± 17
	in study	pre study	in study	pre study	in study	
Biologicals tota	34	3	11	4	8	0
anti-TNF	21	2 (1 × infliximab, 1 × adalimumab)*	11 (adalimumab)	3 (2 × infliximab, 1 × adalimumab)*	8 (adalimumab)	0
anti-IL12/23	10	4	0	2	0	0
anti-Integrin	3	0	0	0	0	0
small molecule	0	0	0	0	0	0
immune suppression	10	5	6	6	0	0
no current therapy	10	0	0	1	0	0

The table outlines the characteristics of patients included in the study.

*These patients had no anti-TNF therapy for at least 4 weeks before the start of the adalimumab study. The reasons for the anti-TNF therapy termination of these patients were therapy failure, severe side effects or personal reasons.

**Table 2.** Clinical parameters of CD cohort 2 at baseline and after 6 weeks TNF therapy.

	remission patients			no-remission patients		
	average value	std. deviation	Wilcoxon, paired p	average value	std. deviation	Wilcoxon, paired p
HBI baseline	11.4	4.4	0.04	12.4	3.8	0.014
HBI 6 weeks in therapy	3.2	3.2		5	2.9	
CDAI baseline	305	118.2	0.001	308	124	0.016
CDAI 6 weeks in therapy	105	95.3		136	88.2	
Limberg score* baseline	2	1	0.35	2	1	0.9
Limberg score^1*^ 6 weeks in therapy	1.5	1		2	1	
CRP baseline	34	42.4	0.65	26	33.1	0.078
CRP 6 weeks in therapy	7	12.2		7	11.8	

*0: normal bowel wall thickness, preservation of wall layer stratification, and no signal on color Doppler; 1: wall thickening and absent color Doppler signal; 2: wall thickening with spot-like focal increases in vascularity; 3: wall thickening and diffuse stretches of increased mural vascularity; 4: wall thickening with increased color Doppler signal in the bowel wall with extension into the mesentery.

### Identification of a microbiota signature predicting response of CD patients to anti-TNF response

**Figure 6.**
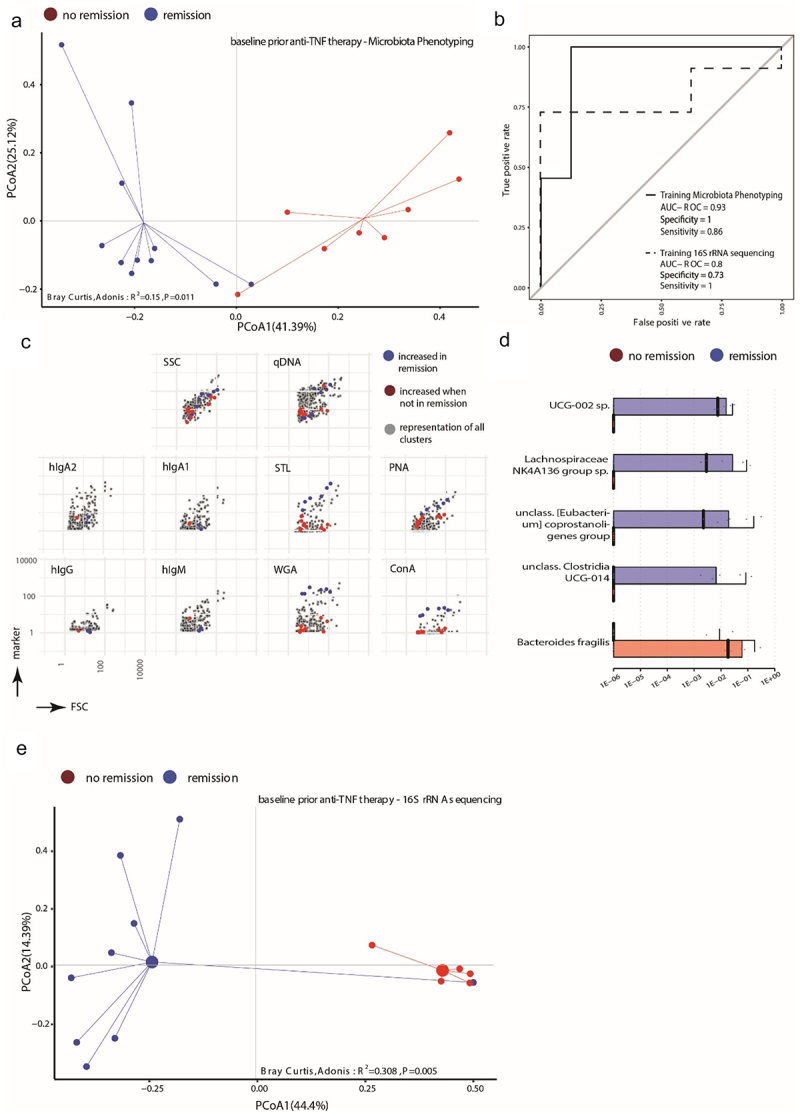
The baseline microbial signature can predict the achievement of remission induced by anti-tnf therapy in Crohn’s disease patients. (a) Principal coordinate projection representing the Bray-Curtis dissimilarity between patients at baseline before initiation of adalimumab treatment stratified into those reaching remission (blue) and those not-fulfilling remission criteria (red) according to 18 cluster selected from the baseline samples. (b) A random forest model classifier to stratify CD patients at baseline into those reaching remission and patients not reaching remission after 6 weeks of adalimumab treatment was trained with 18 selected cluster (solid line) or with 5 taxa (dotted line) identified by 16S rRNA amplicon sequencing. The performance of the respective classifier model is illustrated by the AUROC curves. (c) Projection of the selected clusters containing increased abundance of bacterial cells in CD patients reaching remission or not of the baseline analysis visualizing the mean fluorescence intensity in each stained parameter in relation to all clusters. (d) Abundance of the bacterial taxa significantly differentially abundant between patients reaching remission or not before initiation of adalimumab treatment. Indicated are the median abundance, 95% confidence interval and coefficient of variation. (e) Principal coordinate projection representing the Bray-Curtis dissimilarity between samples of remission (blue) and no remission (red) according to the 5 taxa at baseline before initiation of adalimumab treatment.

We tested whether the microbiota profiles could distinguish therapy-induced remission already at baseline, prior to advent of therapy. For this, the baseline samples were retrospectively categorized according to the ground truth and subjected to the corresponding machine-learning-based feature selection resulting in 18 clusters and significant Bray-Curtis dissimilarity (R^2^ = 0.15, *p* = 0.011, Figure 6a). With the selected 18 clusters, random-forest classification achieved an AUROC = 0.93 (specificity = 1, sensitivity = 0.86) (Figure 6b, solid line). Among the 18 clusters, three were derived from the immunoglobulin panel and 15 from the lectin panel (Supplementary Figure S10A). The clusters predicting remission corresponded to bacterial cells displaying increased staining with all four lectins, while no-remission was predicted by clusters lacking the target sugar moieties of the used lectins (N-Acetyl glucosamine, lactose, mannose) (Figure 6c, Supplementary Figure S10A). At the 6 weeks’ time point, the predictive biosignature was lost and no longer separated remission from no-remission patients (Supplementary Figure S10B, R^2^ = 0.083, *p* = 0.133). From the 16S rRNA gene sequencing dataset, we identified five taxa in total, which resulted in a significant separation between remission and no-remission patients (Figure 6d,e, R^2^ = 0.308, *p* = 0.005). *UCG-002 sp*., *Lachnospiraceae NK4A136 group sp*., *unclassified [Eubacterium] coprostanoligenes group* and *unclassified Clostridia UCG-014* were elevated in abundance in the group of patients that achieved remission during anti-TNF therapy 6 weeks later. In 8 out of the 11 patients achieving remission, any combination of two or more of the remission-associated taxa was detectable. The remission-associated taxa were not found in any of the no-remission patients, but also not in 3 of the remission patients. In the no-remission group, *Bacteroides fragilis* was more abundant and detectable in 7 out of the 8 individuals (Figure 6d). The classification based on the selected taxa performed slightly worse than phenotyping (AUROC = 0.8, specificity = 0.73, sensitivity = 1, Figure 6b, dotted line). Combining microbiota phenotyping and microbiome profiling (22 features: 2 taxa and 20 clusters) increased the Bray-Curtis dissimilarity between remission and no-remission (Supplementary Figure S8B, R^2^ = 0.237, *p* = 0.003). Our data thus indicate that the microbial signature has the potential to predict the response to anti-TNF therapy in CD patients.

## Discussion

In this study, we describe the phenotypic characterization of single bacterial cells in complex intestinal communities by multi-parameter microbiota flow cytometry (mMFC) motivated by the observation that immunoglobulin-coating and lectin-binding to intestinal microbiota implied taxonomically dependent and independent information correlating to CD. The IgA-coated fractions of the intestinal microbiota were taxonomically significantly different between CD patients and healthy controls, and were representative of the overall microbiome composition of both groups (Figure 1a,d). The lectin-stained fractions, both WGA to PNA, show a decreasingly distinct profile between CD and healthy controls (Figure 1b,c) and enriched some of the CD-related potentially colitogenic taxa but also taxa that were not visibly different between the cohorts in the total microbiome (Supp. 3A,B). Overall we hypothesize that in disease, in this case CD, the intestinal microbiota adapts to the altered condition of the host, which is reflected by the altered patterns of immunoglobulin coating and surface sugar expression.

To explore this, we simultaneously analyzed quantitative DNA staining, light scattering, host-immunoglobulin coating, and expression of certain sugar residues on the surface of the bacteria. Using dimensionality reduction, machine learning for feature selection and random forest modeling for automated sample classification, we demonstrate the feasibility of using single-cell-based microbiota characterization to identify disease-specific biosignatures for patient stratification and monitoring. The mMFC-based microbiota phenotyping reliably classified CD patients and healthy donors. Thus, in addition to the microbiome alterations on the taxonomic level described for CD, we here show that surface characteristics of bacteria are presumably impacted by the inflammatory environment in CD and distinct from healthy controls.

Our results indicate that mMFC could be an effective method alternative to or complementing the wide-spread sequencing approaches, for microbiome-based diagnostics and the analyses of disease- and therapy-specific alterations.^13^ The markers used in this study were suitable to differentiate CD patients from healthy individuals and to stratify CD patients. The inclusion of other markers may help or be necessary for the discrimination of other diseases. Technically, the mMFC approach maintains the flexibility to easily adapt the staining panel accordingly when required. We suspect that the phenotypic features, we assess by mMFC in high granularity, reflect adaptions of the immune system, the host-microbiome interaction and the bacteria and are less sensitive to taxonomic variations between individuals. Although both CD patients and healthy individuals have IgA-coated bacteria in their microbiota, the highly resolved phenotypic differentiation of the bacterial phenotype may allow to discriminate the homeostatic state from the disease-related state of IgA coating,^28^ confirming that immunoglobulin coating of microbes in IBD identifies colitogenic, potentially pathogenic bacteria representative for the taxonomic dysbiosis.^7,9,29^ In a cohort of 19 patients with CD receiving anti-TNF therapy, we observed that remission of CD patients induced by anti-TNF therapy was primarily reflected by changes in bacteria coated with host immunoglobulins, perhaps indicating an alteration of the mucosal humoral immune response by therapy. This is in line with previous findings that during anti-TNF therapy the expression and secretion of mucosal antibodies is changing.^29,30^ Taxonomically, we observed that *Coprococcus comes* positively and Bacteroides species negatively correlated with anti-TNF-induced remission. For *Coprococcus* this has been observed before.^31^

Changes in functionality rather than taxonomy have been shown to better characterize the dysbiosis in IBD.^32^ Correspondingly, we have observed that altered lectin-binding patterns do not correlate to altered taxonomic composition of the overall microbiome but have relevant impact on CD-specific microbiota phenotype signatures, suggesting that altered lectin-binding indicates cellular adaptions of certain bacteria rather than compositional changes of the community. Accordingly, we have observed that the staining with lectins is different in axenic cultures depending on growth state, pH and medium composition (data not shown). For the initial prediction of the remission potential of individual patients to anti-TNF therapy prior to therapy, the expression of sugar moieties of individual bacteria was very predictive, while surface coating with immunoglobulins was not relevant for the prediction of therapy response. Previous findings that the metabolic capacity of a microbiome and metabolite exchange between bacteria correlates with a positive therapy response^31^ support our assumption that the single-cell resolved analysis of surface sugars mirrors metabolic activity and intercellular crosstalk. The presence of a phenotypic signature that predicted anti-TNF-induced remission before start of the therapy highlights the potential of mMFC for patient stratification. However, further studies will be needed to clarify the molecular link between surface sugars and metabolism. The taxonomic profiling identified *UCG-002 sp., Lachnospiraceae NK4A136 group sp., unclass. Clostridia UCG-014* and *unclass. Eubacterium coprostanoligenes group* which, when detected at baseline, predicted a positive therapy outcome, nevertheless the classification performance by a random forest model was limited. In such a scenario, we showed, that mMFC and 16S rRNA sequencing could complement each other to increase predictive power.

While at this point we cannot draw conclusions about any causal relationship between microbiota phenotype and disease pathogenesis *per se*, the flow cytometric approach offers the tools to do so, by giving access to the bacteria with a particular phenotype directly by cell sorting,^33^ although adaptations in the technology need to be considered to gain access to strictly anaerobic bacteria.^34^

In conclusion, we here demonstrate that single-cell phenotyping of bacteria by multi-parametric microbiota flow cytometry (mMFC) is a potent tool for the investigation and characterization of complex microbial communities, such as the intestinal microbiota, for the identification of disease-specific bacterial signatures and generates robust results compared to 16S rRNA sequencing. Additionally, mMFC captures parameters e.g. sugar-moiety composition that were previously neglected in microbiome profiling but allowed for prediction of therapy outcome. Considering the widespread use of flow cytometry in diagnostics, mMFC presents a viable alternative and supplementation to “conventional” microbiota profiling with the potential to be applied for point-of-care diagnostics and therapy monitoring, but also to investigate the role of defined bacteria in disease pathogenesis.

## Material and methods

### Stool samples

Stool samples were provided by all donors under approval of the local ethics committee of the Charité Berlin (approval reference: EA4/014/20; EA4/247/20) and in accordance to the Helsinki II Declaration. The samples were taken with stool sampling tubes (Sterilin®, VWR Cat. No. 215–0327) and transferred to 4°C instantly upon arrival.

#### Patient and public involvement

During patient recruitment, the study was openly and transparently communicated by informed patient consent form. Processed study data was made available at all stages of the study to the participants. Healthy controls completed a questionnaire assessing potential confounders such as age, gender, smoking status, and nutritional habits. The control group did not report any chronic intestinal inflammation. The patients’ disease state and entity were indicated by the examining clinicians. The sample collection logistics was designed to enable easy participation in the study, e. g. from home. Members of the research team are actively involved in science communication events to explain the methods and goals of this study to the broad public and to receive feedback regarding patients’ needs and concerns.

### Cohorts

For this study two independent CD cohorts were recruited (characteristics summary in Table 1). Cohort 1 (*n* = 57) was recruited from the IBD ambulance ward at Charité-Universitätsmedizin Berlin (CCM) without selection of the patients in regards to inflammation severity, disease duration or therapy. This cohort laid the groundwork to characterize the microbiota of CD patients and was applied in the comparisons to healthy controls (*n* = 44, age- and sex-matched, Table 1) to define the CD biosignature for each of the comparisons (training sets). Cohort 2 consisted of CD patients (*n* = 19) who participated in an independent study that investigated the predictability of TNF-therapy efficacy. The patients of cohort 2 were selected for active disease (HBI >5) and samples were collected prior to anti-TNF therapy (CD cohort 2 baseline) as well as after 6 weeks of therapy (CD cohort 2 therapy). Cohort 2 was applied to validate the biosignatures of CD when compared to healthy controls (*n* = 10) which were not originally used for model training, and to define new biosignatures to predict and evaluate the success of anti-TNF-therapy in these patients.

#### Stool sample processing

Stool samples were kept at 4°C for a maximum of 96 h before processing. When longer storage was required, samples were frozen directly at −20°C or −80°C. Each sample was diluted in autoclaved and 0.2 µm sterile-filtered PBS (in-house, Steritop® Millipore Express®PLUS 0.22 µm, Cat. No: 2GPT05RE) to a concentration of 100 mg/ml and homogenized by vortexing. The suspension was sequentially filtered through 70 µm (Falcon, Cat. No. 352350) and 30 µm filters (CellTrics®, Sysmex, Cat. No. 04–0042–2316). 10 µl were stored directly at −20°C for 16S rRNA gene sequencing. For each sample stocks at OD = 0.4 determined at 690 nm with a VIS-spectrometer (Multiskan™ FC, Thermo Scientific™) were prepared for long-term storage by re-suspending the respective volume from the microbiota suspension in 1 ml 40% glycerol/LB freezing medium and immediate transfer to-80°C.

#### Staining microbiota for multi-parameter microbiota flow cytometry (mMFC)^21^

Frozen microbiota stocks (OD 0.4) were topped up with 1 mL of autoclaved and sterile-filtered PBS and centrifuged at 13,000 × g for 10 min, 4°C. The pellet was incubated in 500 µl blocking solution containing 20 µg/ml mIgG1 (clone: IS5-21F5, Miltenyi Biotech Cat. No.: 130-106-545) and 10 µg/ml mIgG2a (clone: S43.10, Miltenyi Biotech Cat. No.: 130-106-546) in PBS for 5 min at RT. The suspension was topped with 1.5 ml PBS and subjected to another centrifugation step (13,000 × g, 10 min, 4°C). The pellets were re-suspended in PBS containing 0.2% BSA (v/w) and 25 µg/µl DNase (Sigma Aldrich Cat. No. 10104159001), which was also used as staining buffer. Cell density was adjusted to 0.02–0.04 OD_690_/ml. 100 µL of the cell suspension was used for one test, e.g. stained with the immunoglobulin panel. The antibodies used were anti-human IgM-Brilliant Violet 650 (clone: MHM-88, Biolegend® Cat. No. 314526), anti-human IgG-PE/Dazzle™ 594 (clone: HP6017, Biolegend® Cat. No. 409324), anti-human IgA1-Alexa Fluor 647 (clone: B3506B4, Southern Biotech Cat. No. 9130–31), anti-human IgA2-Alexa Fluor 488 (clone: A9604D2, Southern Biotech Cat. No. 9140–30). The lectins used for staining were 0.5 µg/test Peanut Agglutinin-CF®488 (PNA, Biotium Cat. No.29060), 0.5 µg/test Concanavalin A-CF®680 (Con A, Biotium Cat. No. 29020–1) and 0.25 µg/test Wheat Germ Agglutinin-CF®555 (WGA, Biotium Cat. No.29076–1); 0.5 µg/test of biotinylated Solanum Tuberosum Agglutinin (STL, Vector Laboratories/Biozol Cat. No. B-1165) were shortly pre-incubated with 2 µL (1:50, v/v) anti-Biotin-PerCP antibody (clone: Bio3-18E7, Miltenyi Biotech Cat. No. 130-133-293) before adding to the residual reagents. The tests were incubated for 30 min at 4°C and subsequently topped up with 1 ml 5 µM Hoechst solution (Hoechst 33,342, Thermo Fisher Scientific Cat. No. 62249) for another 30 min at 4°C. After incubation the tests were washed with 900 µl PBS/BSA at 13,000 × g and re-suspended in fresh PBS/BSA for acquisition.

### Microbiota flow cytometry

BD Influx® cell sorter was used for all cytometric measurements. The sheath buffer (PBS) for the instrument was autoclaved and sterile filtered (Steritop® Millipore Express®PLUS 0.22 µm, Cat. No: 2GPT05RE) before each fluidics start up. The quality and reproducibility of each acquisition was controlled by the alignment of lasers, laser delays and laser intensities by Sphero™ Rainbow Particles (BD Biosciences Cat. No. 559123) and control of scatter properties by Megamix-Plus FSC beads (BioCytex Cat. No7802). Samples were acquired with an event rate below 15,000 events/second. For each sample, 3 × 10^5^ Hoechst 33,342-stained events (mean fluorescence intensity > 10) were recorded. We controlled the staining procedure throughout the study by including a standardized microbiota sample (anchor sample) comprising a pool of different donors.

#### Graphical and statistical data analysis

Statistical analyses were implemented through R (v. 4.0.3 or later versions, Figure 2c), unless stated otherwise (supplementary Methods). Computation of β-diversity with Bray-Curtis dissimilarity was computed using vegdist(data, method=”bray”) function from vegan package.^35^ The Bray-Curtis dissimilarity is a statistical metric to quantify the difference in composition between two cohorts. In our case it is the abundance of cells in each cluster or the presence or absence of bacterial taxa and their abundance. The Bray-Curtis dissimilarity is defined by a number between 0 and 1, 0 indicating that two samples/cohorts are completely similar and 1 indicating that two samples/cohorts do not share anything. The PCoA was computed by the R base function cmdscale() on the respective distance matrix followed by Adonis test adonis() from vegan package to evaluate the variance within groups. Graphical representation of the dissimilarity of all samples by Principal Coordinates Analysis (PCoA) was plotted using ggplot2 package. Correlation analyses data was evaluated by Pearson’s r e. g. in ggscatter([…], cor.method=”pearson”) and the statistical comparisons of paired data points by stat_compare_means() using ggpubr package.

#### Clustering of flow cytometric data

The outline of the computational steps are shown in Figure 2c. Raw FCS-files from flow cytometry were imported to R without any transformation using FlowCore’s read.flowSet()^36^ and was gated in the environment of the flowWorkspace^37^ environment to reduce instrument noise by including only events > 1 for forward (FSC) and side scatter (SSC). A self-organizing map (SOM, kohonen package)^38,39^ was previously trained on a data set comprising 3 × 10^5^ cells subsampled and concatenated from approx. 300 samples (comprising a mixture of patients with different chronic inflammatory diseases and healthy controls) stained for the immunoglobulin coating and the surface sugar expression to capture a widespread diversity of mMFC patterns by the som() function of the kohonen package for a 5 × 5hexagonal map describing the data based on gaussian neighborhood. For SOM training on the 3 × 10^5^ cells subsample clusters should contain at least 0.05% (i.e. 150 cells) of the cells which yielded 2025 cluster for each staining panel. To characterize the phenotypic diversity of the samples used in this study, we then mapped the pre-determined cluster to each sample by kohonen:map() after downsampling to 3 × 10^4^ cells per individual in favor of computing power and time, as we found 3 × 10^4^ cell to represent a sample sufficiently. Each sample is now described by an individual abundance of cells in each cluster. To assess the information of immunoglobulin coating and surface sugar moieties presence simultaneously we combine both cluster sets. Thus the same bacteria are described by two different SOMs and the number of clusters adds up to 4050 which we termed the microbiota phenotype.

#### Feature selection & modelling

The outline of the computational steps is shown in Figure 2c. Cellular frequencies within each mMFC cluster and relative abundances of bacterial genera, respectively, were used as inputs for random forest machine learning models for sample classification. Briefly, all data were pre-filtered to exclude non-significant features with *p* > 0.05, Wilcoxon rank-sum test (vegan package,^35^ removing all mMFC clusters and 16S rRNA sequencing-derived taxonomic units not contributing to the discrimination between the cohorts. In a second filtering step, Recursive Feature Elimination (RFE) with 10-fold cross-validation was applied to remove weak features for classification, with rfeControl() function in caret package.^40^ The importance of the selected features was obtained within the RFE according to the consensus ranking through the 10-fold cross-validation. The resulting features were used to train the random forest model involving 10 times repeated 10-fold cross-validation with function of train() and trainControl(), to mitigate overfitting (caret and MLeval package).^40,41^ 10 randomly selected samples each from the healthy controls and all samples from CD cohort 2 originally excluded from all steps of feature selection were used to evaluate the models’ predictive performance in one modeling attempt. All procedures related to model construction were performed in caret package,^40^ and evaluation of predictive ability (performance metrics, AUROC, sensitivity, specificity, and confusion matrix at default threshold 0.5) was implemented using MLeval package.^41^

#### Fluorescence-activated cell sorting of bacteria

For cell sorting, the BD Influx cell sorter was used. The event rate was adjusted to below 10^4^ events/sec. The drop delay and deflection accuracy was set with Accudrop (BD Biosciences, Cat No. 345249). The sorter was operated with 1-drop pure settings. A minimum of 10^5^ cells per sample and phenotype were sorted into buffer-pre-coated 2 ml reaction tubes. Samples were stored on ice until the complete sort was finished. The fractions were then spun down for 15 min at 17,000 × g, the supernatant was removed and the cells left in a volume of approx. 50 µl were stored at −20°C until further processing.

#### 16S rRNA sequencing (illumina MiSeq platform)

For 16S rRNA gene sequencing, we amplified the V3/V4 region of the 16S rRNA gene (for: TCGTCGGCAGCGTCAGATGTGTATAAGAGACAGCCTACGGGnGGCWGCAG, rev: GTCTCGTGGGCTCGGAGATGTGTATAAGAGACAGGACTACHVGGGTATCTAATCC^42^; TIB MOLBIOL Syntheselabor GmbH) directly from a microbiome sample with a prolonged initial heating step of 5 min. After the amplicon PCR the genomic DNA was removed by AmPure XP Beads (Beckman Coulter Life Science Cat. No. A63881) with a 1:1.25 ratio of sample to beads (v/v). The amplicons were checked for their size and purity on a 1.5% agarose gel, and if suitable, subjected to the index PCR using the Nextera XT Index Kit v2 Set C (Illumina, FC-131-2003). After Index-PCR, the samples were cleaned again with AmPure XP Beads (Beckman Coulter Life Science Cat. No. A63881) in a 1:0.8 ratio of sample to beads (v/v). Samples were analyzed by capillary gel electrophoresis (Agilent Fragment Analyser 5200) for correct size and purity with the NGS standard sensitivity fragment analysis kit (Agilent Cat. No. DF-473). Of all suitable samples a pool of 2 nM was generated and loaded to the Illumina MiSeq 2500 system.

#### Full-length 16S rRNA sequencing (PacBio platform)

The PacBio 16S rRNA sequencing protocol was conducted as described by PacBio’s instructions for the SMRTbell® Express Template PrepKit 2.0 (Pacific Biosciences of California, Inc; Cat. No. 101-685-400). Briefly, bacterial full-length 16S rRNA gene was amplified with barcoded primers (fwd.: AGRGTTYGATYMTGGCTCAG; rev.: RGYTACCTTGTTACGACTTT). 5 μL from each sorted sample were used as input DNA. After successful amplification, 500 ng of each barcoded amplicon were pooled for library construction. Before library construction the quality of the pooled input DNA was control by capillary gel electrophoresis (Agilent Fragment Analyser 5200), followed by cleanup from non-PCR product by AmPure XP Beads (Beckman Coulter Life Science Cat. No. A63881). Afterwards, DNA repair and A-tailing as well as adapter ligation with subsequent bead purification were performed according to the PacBio protocol. The prepared library was subjected to full-length 16S rRNA sequencing by the PacBio Sequel I system using SMRT Link Version 8 including sequencing primer annealing and polymerase binding.

### Sequence alignment illumina MiSeq data

Paired-end reads generated by Illumina MiSeq 16S rDNA sequencing were filtered and trimmed using Trimmomactic (Version 0.39).^43^ 7 leading bases with qualities below 35 were trimmed and reads shorter than 180 bases were filtered out. Using the DADA2 (Version 1.22.0) software package,^44^ forward and reverse reads were truncated at 260 and 210 bases respectively and filtered with a minimum quality score of 12 and a maximum of 0 ambiguous nucleotides. Amplicon sequence variants (ASVs) were identified using the default settings of the DADA2 algorithm and ASVs were classified using the Silva 138.1 prokaryotic SSU taxonomic training data formatted for DADA2.^45^ After alignment of the sequences with DECIPHER (Version 2.24.0),^46^ a phylogenetic tree was computed using FastTree (Version 2.1.11).^47^ For analysis the ASVs were matched with the respective phylogenetic information, the data was processed to genus level and normalized prior to any further calculations.

### Sequence alignment PacBio SMRT link data

After sequencing, samples were demultiplexed and circular consensus sequence (CCS) reads were constructed using PacBio’s software followed by filtering with a 99% base call precision (quality score of 20). Further processing was done using the DADA2 pipeline (version 1.24.0) functions. First, sequencing primers were removed and reads without primers were discarded. Unique sequences were then identified and dereplicated, i.e. collapsed into a set of clustered ASVs. After learning and removing errors from amplicon reads, the ASV counts observed in each sample were recorded in a summary table. Chimeras were removed from the sequences and taxonomy assignments to ASVs were achieved by implementing the Ribosomal Database Project naive Bayesian classifier algorithm as described by.^48^ Species-level annotation were added to taxonomic assignments by exact matching against the Silva 138.1 reference FASTA database.^49^ For analysis the ASVs were matched with the respective phylogenetic information, the data was processed to genus level and normalized prior to any further calculations.

## Abbreviations

CD: Crohn’s disease
mMFC: multi-parameter microbiota flow cytometry
PCoA: principal coordinate analysis
SOM: self-organizing map
AUROC: area under the receiver operator curve
RFE: recursive feature elimination
HBI: Harvey-Bradshaw index

## Supplementary Material

Supp figures.docx

## Data Availability

10.5281/zenodo.13375351
