# Supplementary material for: Single-cell microbiota phenotyping reveals distinct disease and therapy-associated signatures in Crohn’s disease
Source: Gut Microbes. 2025 Jan 15;17(1):2452250. doi: 10.1080/19490976.2025.2452250 (PMC11740678; doi:10.1080/19490976.2025.2452250)
Supplement: Supp figures.docx [file KGMI_A_2452250_SM6627.docx]

# Supplemental Figure Legends

**
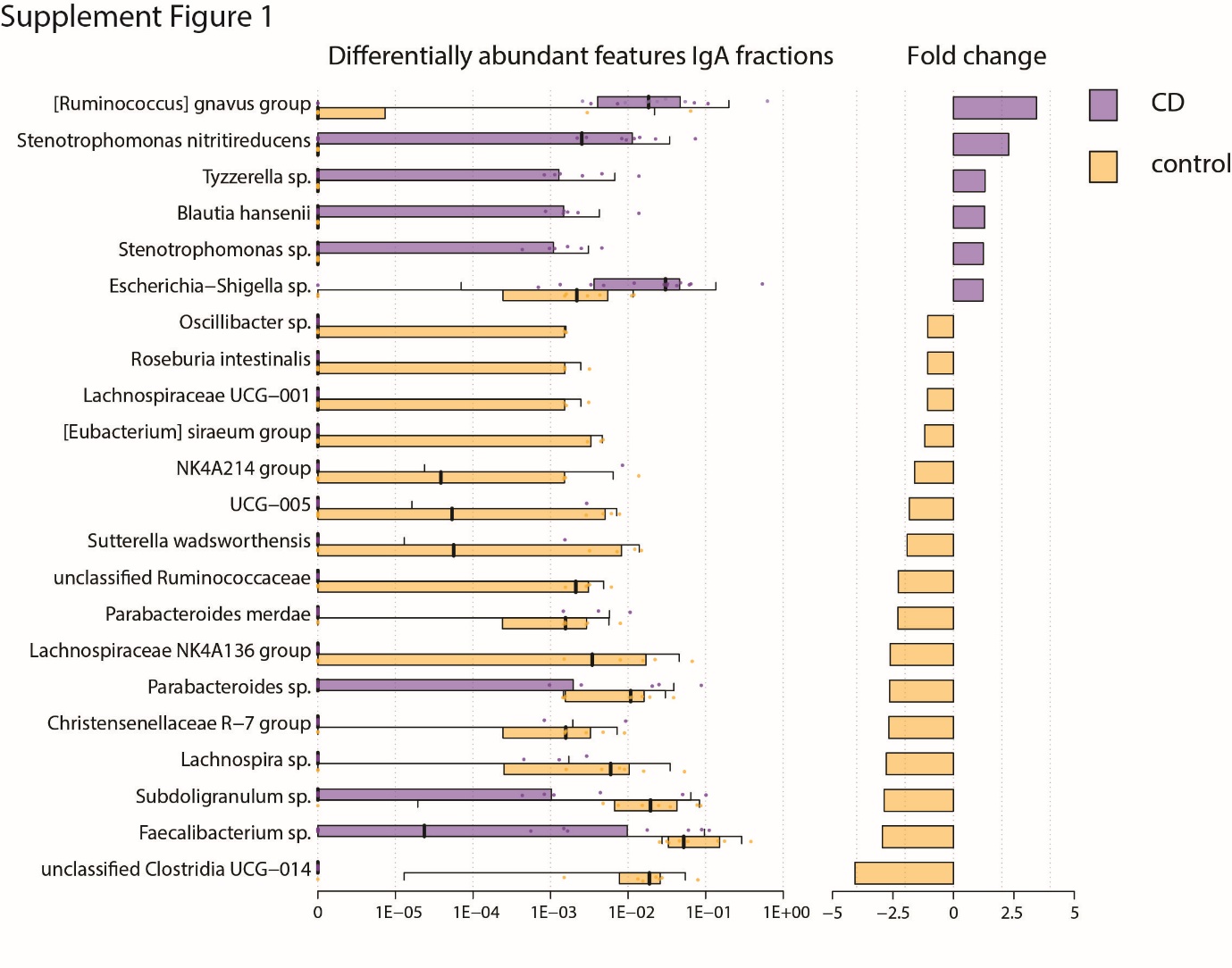
Supplemental Figure 1. Differences in the composition of bacteria sorted according to host-IgA-coating between CD patients and healthy donors.** Stool samples of selected CD patients (n=14) and healthy donors (n=8) were stained for human IgA1/2 coating, sorted by FACS and analysed by full-length 16S rRNA gene sequencing. The abundance of each taxon in each sorted sample is indicated. Shown is the mean abundance, 95% confidence interval and coefficient of variation. In addition, the fold change in mean abundance of the respective taxon between the IgA-coated fraction of the CD patients and healthy donors is indicated.

**
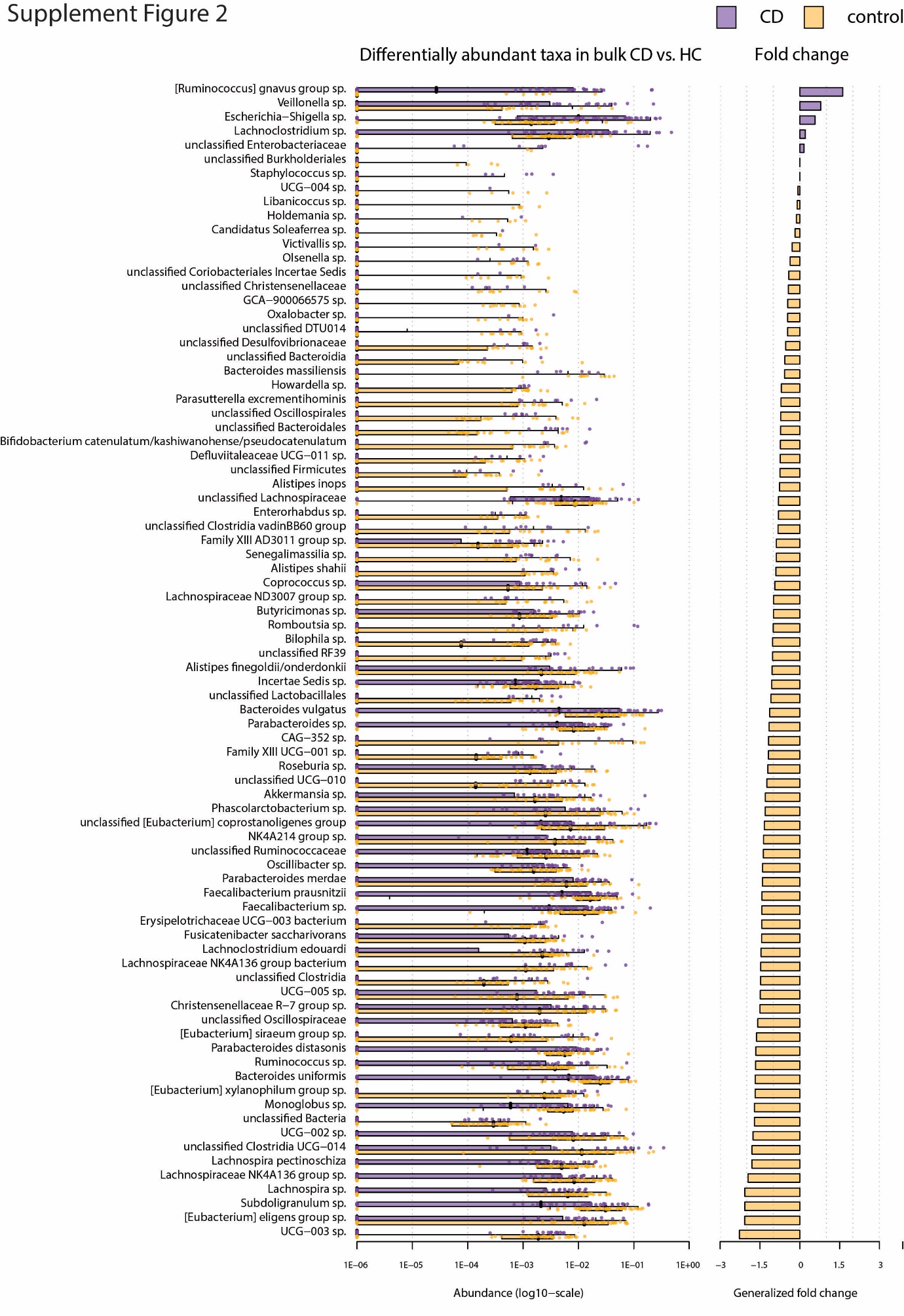
Supplemental Figure 2. Differences in the composition of the intestinal microbiome between CD patients and healthy donors.** The entire microbiome of CD patients (CD cohort 1, n=55) and healthy controls (n=44) were analysed by 16S rRNA sequencing. The data was then investigated for statistically different taxa between these groups and those further refined by RFE yielding a list of 82 taxa. The abundance of each taxon in each sorted sample is indicated. Shown is the mean abundance, 95% confidence interval and coefficient of variation. In addition, the fold change in mean abundance of the respective taxon between the IgA-coated fraction of the CD patients and healthy donors is indicated.

**
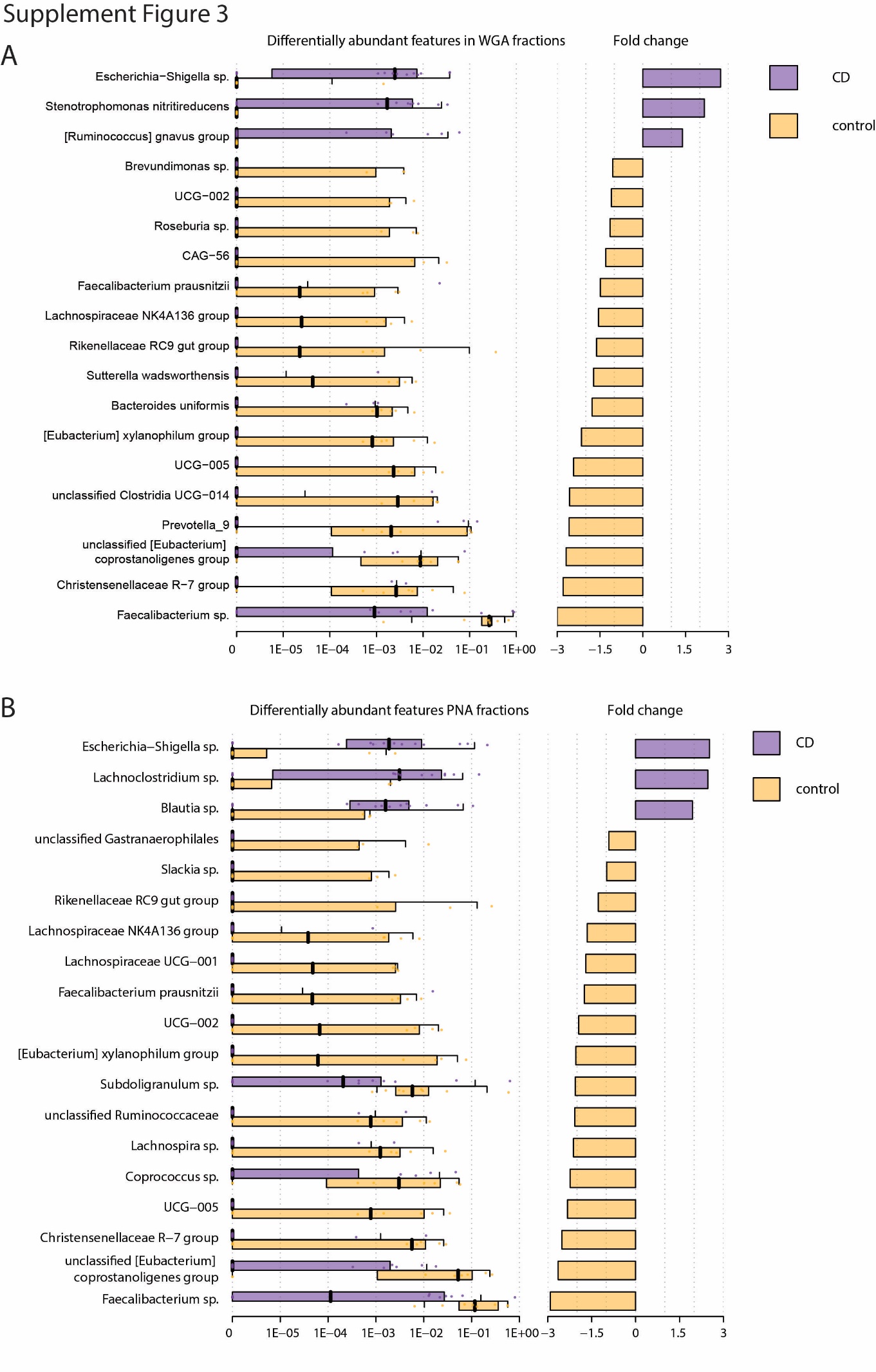
Supplemental Figure 3. Differences in the composition of bacteria sorted according to lectin staining between CD patients and healthy donors.** Stool samples of selected CD patients (n=14) and healthy donors (n=8) were stained for **(A)** peanut agglutinin (PNA) or **(B)** wheat germ agglutinin (WGA), sorted by FACS and analysed by full-length 16S rRNA gene sequencing. The abundance of each taxon in each sorted sample is indicated. Shown is the mean abundance, 95% confidence interval and coefficient of variation. In addition, the fold change in mean abundance of the respective taxon between the lectin stained fractions of the CD patients and healthy donors is indicated.

**
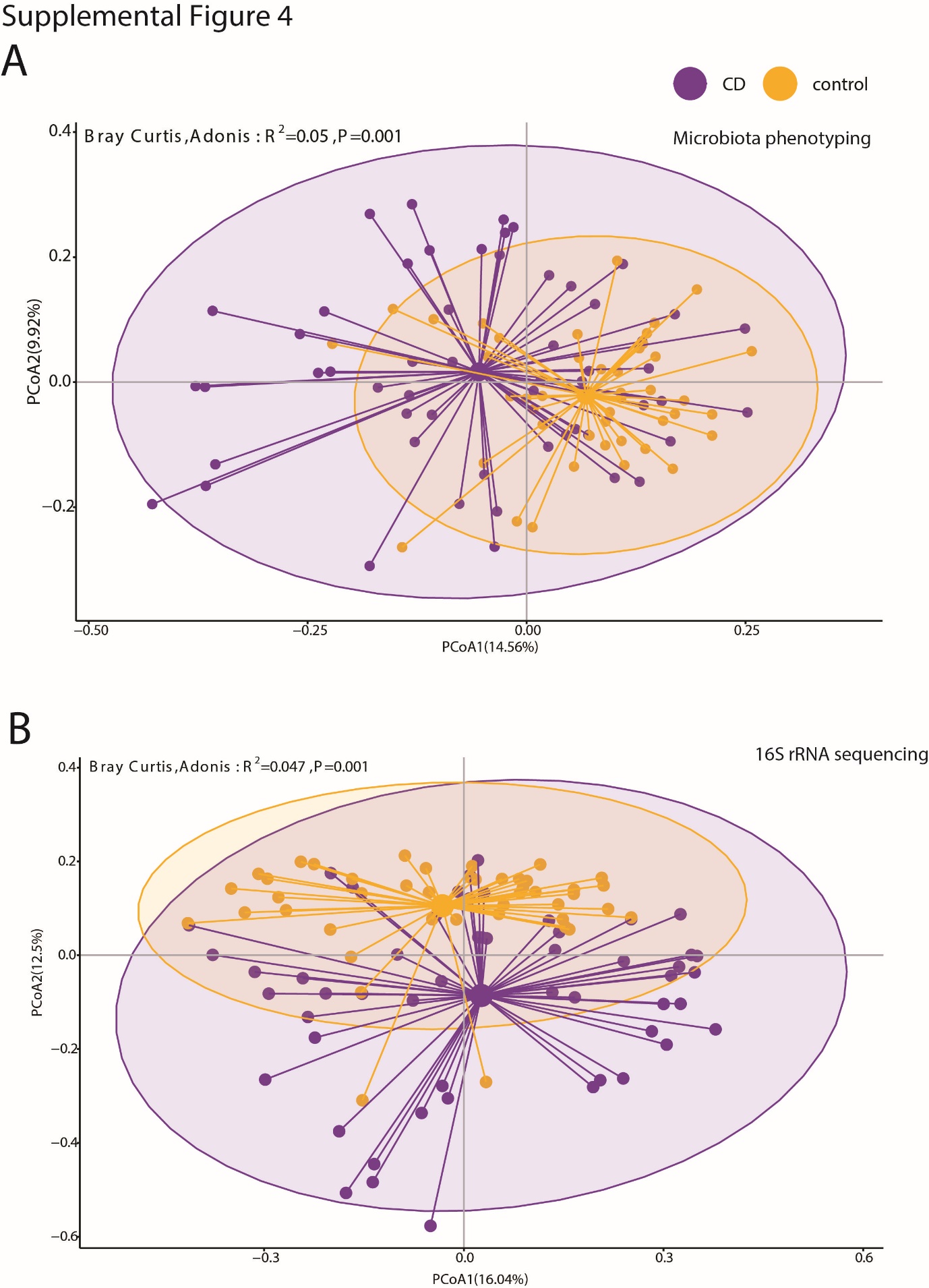
Supplemental Figure 4. The microbial signature of Crohn’s disease patients and healthy controls.** Samples of CD patients from cohort 1 (n=55) and healthy controls (n=44) were **(A)** stained for host immunoglobulins and surface sugars or **(B)** characterised by 16S rRNA gene amplicon sequencing. The β-diversity (Bray-Curtis dissimilarity) between all samples is represented by principal coordinate projection according to all 4050 phenotypic clusters or all taxa identified.

**
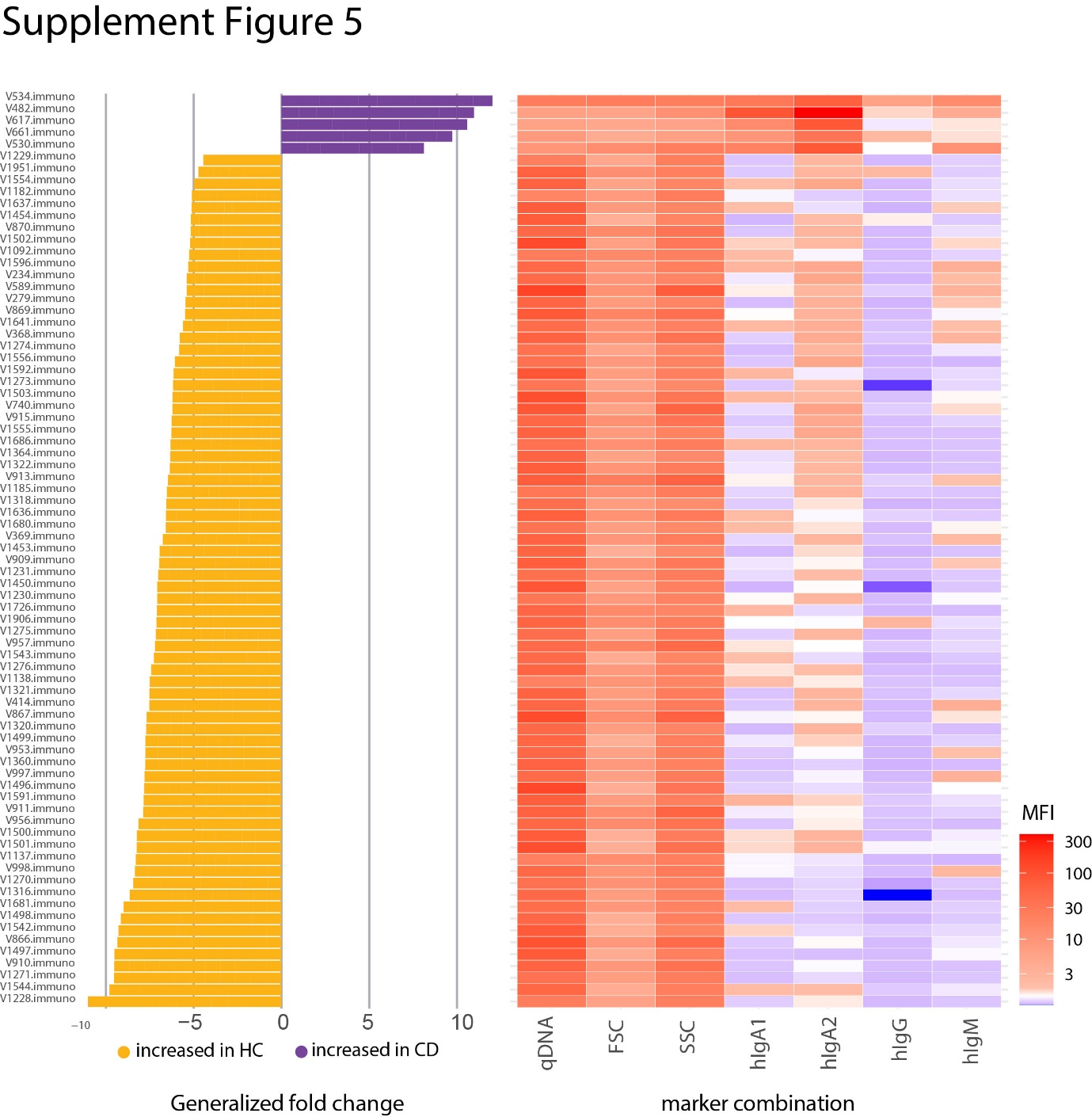
Supplemental Figure 5. Fold change in abundance and characteristics of the selected clusters of the immunoglobulin staining panel within the phenotypic biosignature of CD patients compared to healthy controls.** Representation of the 77 clusters of the immunoglobulin panel selected by Wilcoxon test and recursive feature elimination differentiating between CD patients and healthy controls. Fold change indicates the difference in relative abundance of cells in that cluster between CD and healthy control samples. The heat map represents the phenotype of the cells defining that cluster as mean signal intensity.

**
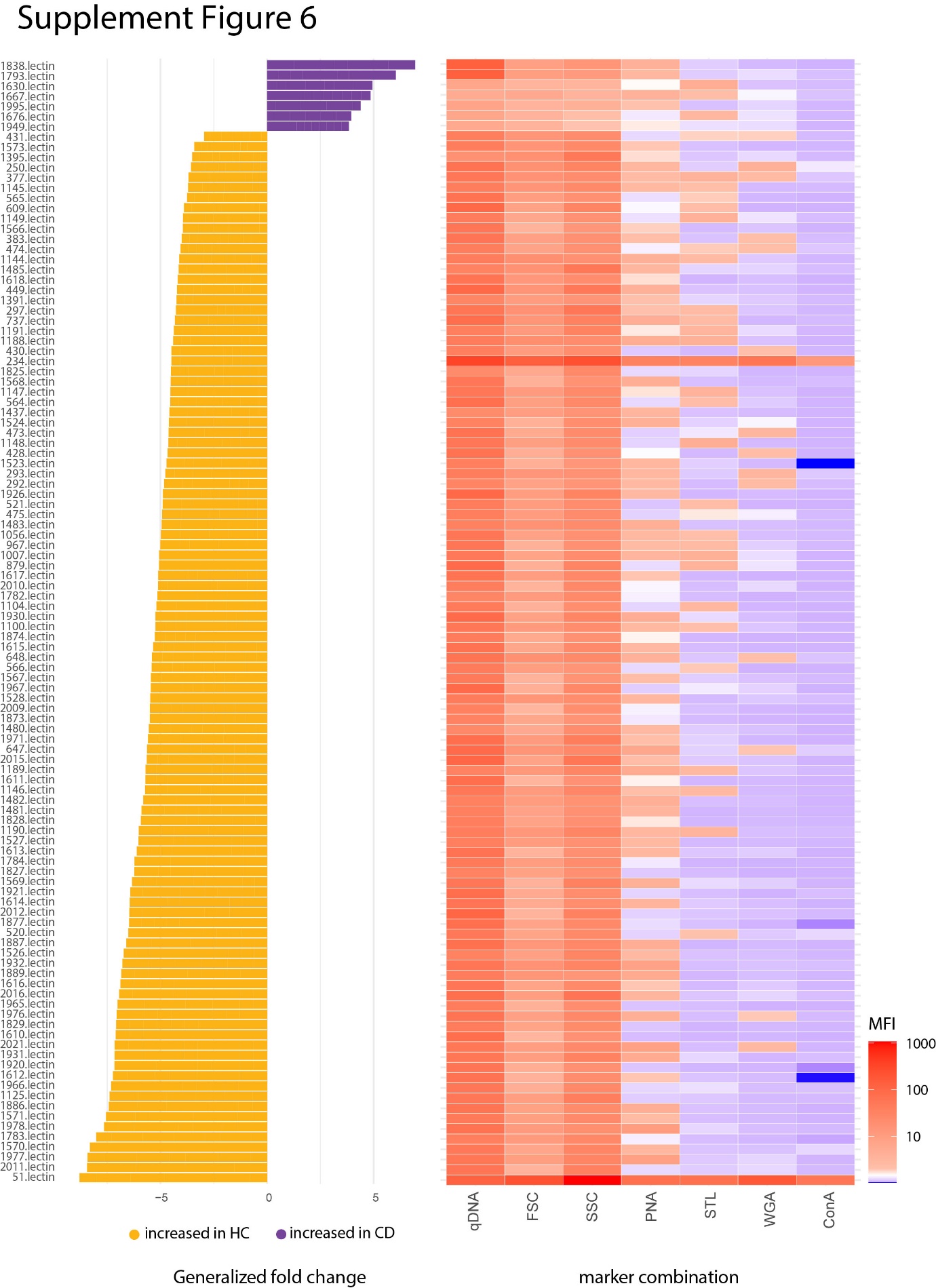
Supplemental Figure 6. Fold change in abundance and characteristics of the selected clusters of the lectin staining panel within the phenotypic biosignature of CD patients compared to healthy controls.** Representation of the 110 clusters of the lectin panel selected by Wilcoxon test and recursive feature elimination differentiating between CD patients and healthy controls. Fold change indicates the difference in relative abundance of cells in that cluster between CD and healthy control samples. The heat map represents the phenotype of the cells defining that cluster as mean signal intensity.

**
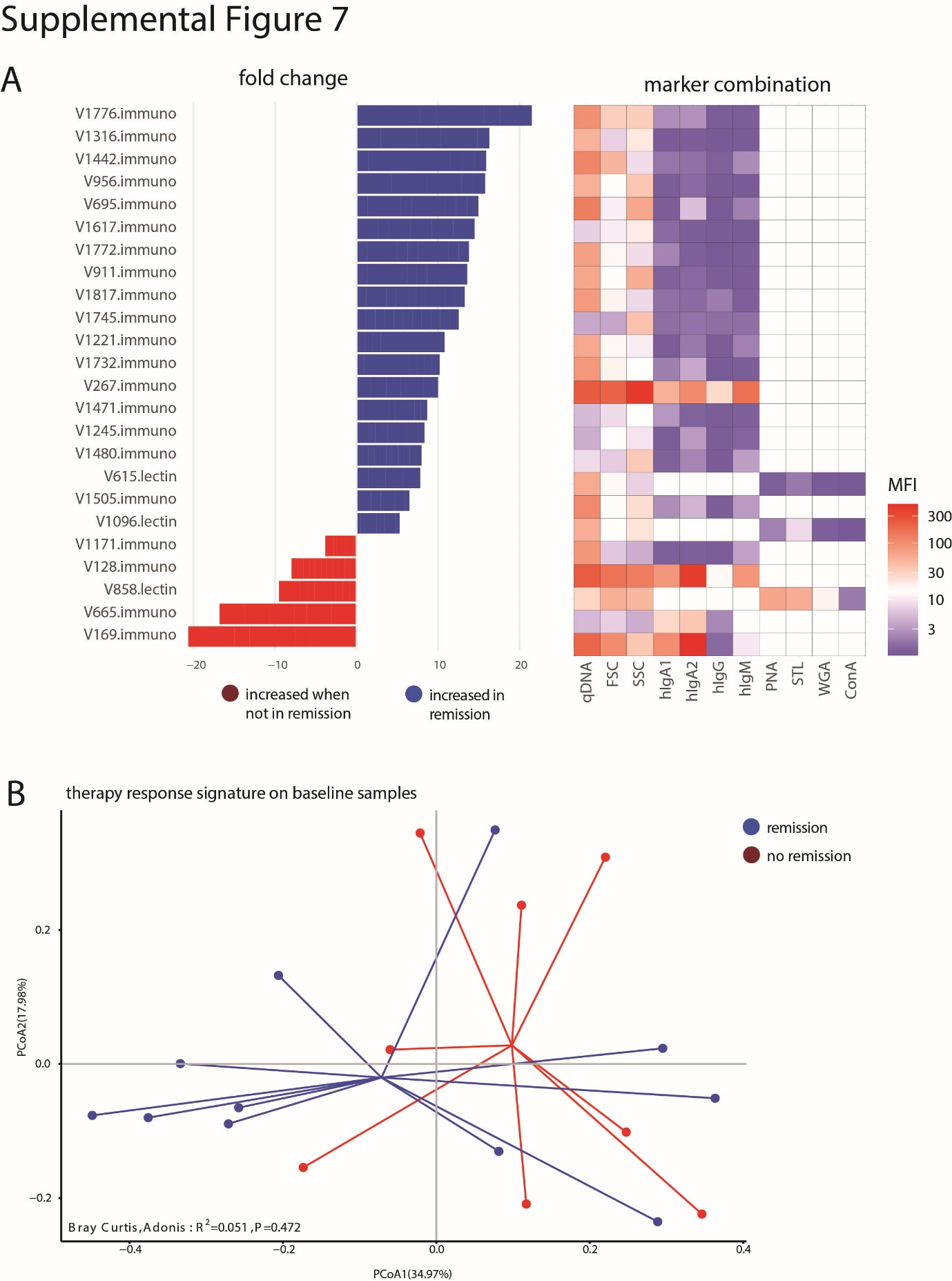
Supplemental Figure 7. The bacterial phenotypic signature correlating with achievement of remission in Crohn’s disease patients after 6 weeks of anti-TNF therapy**. **(A)** Representation of the total 24 clusters of the immunoglobulin and lectin panel selected by Wilcoxon test and recursive feature elimination differentiating between CD patients of cohort 2 which have achieved remission and those which have not reached remission criteria. Fold change indicates the difference in relative abundance of cells in that cluster between remission and no-remission CD patients. The heat map represents the phenotype of the cells defining that cluster as mean signal intensity normalized to the minimal and maximal signal observed in that parameter. **(B)** Principal Coordinate projection representing the Bray-Curtis dissimilarity between remission and no-remission patients at baseline according to the 24 clusters selected to distinguish success of anti-TNF therapy 6 weeks later.

**
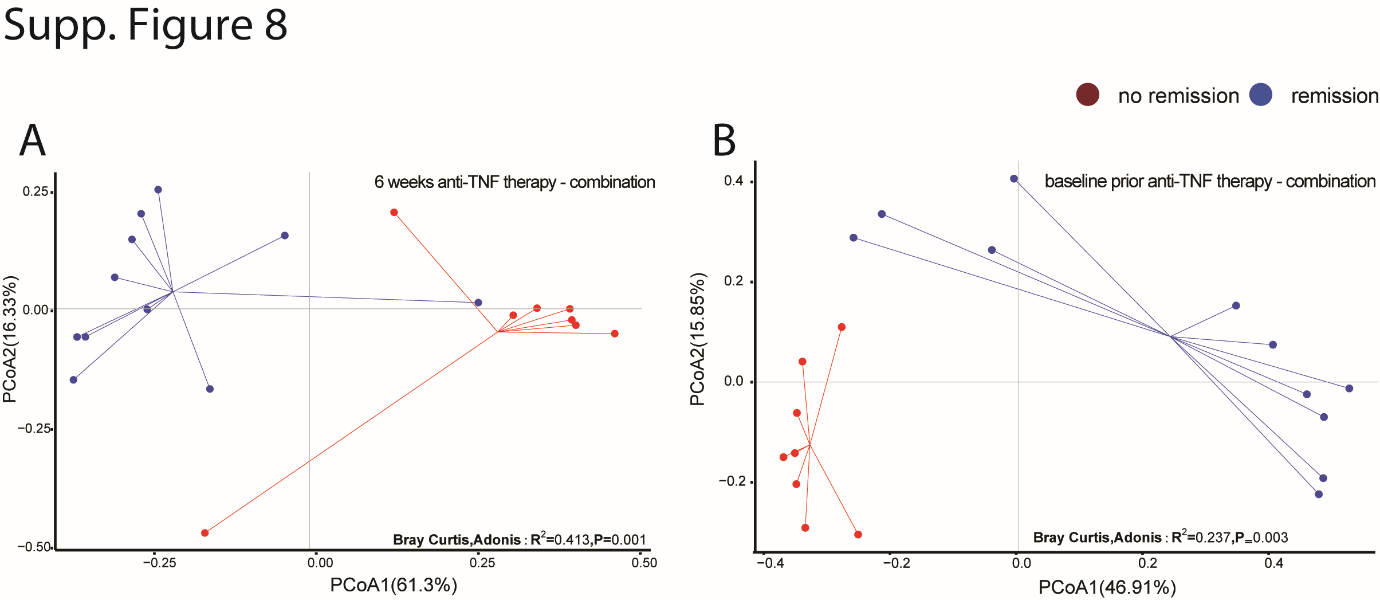
Supplemental Figure 8. Correlation of therapeutic success with anti-TNF therapy with integrated taxonomic and phenotypic signature. (A)** Principal Coordinate projection representing the Bray-Curtis dissimilarity between samples of remission and no-remission patients at baseline before therapy according to the 5 selected taxa shown in Fig. 5D. **(B)** Principal Coordinate projection representing the β-diversity (Bray-Curtis dissimilarity) between samples of remission and no-remission patients at baseline according to Wilcoxon test and recursive feature selection of an integrated dataset comprising the 16S rRNA gene amplicon sequencing and mMFC-based phenotyping. 20 phenotypic clusters and 2 taxa were selected.

**
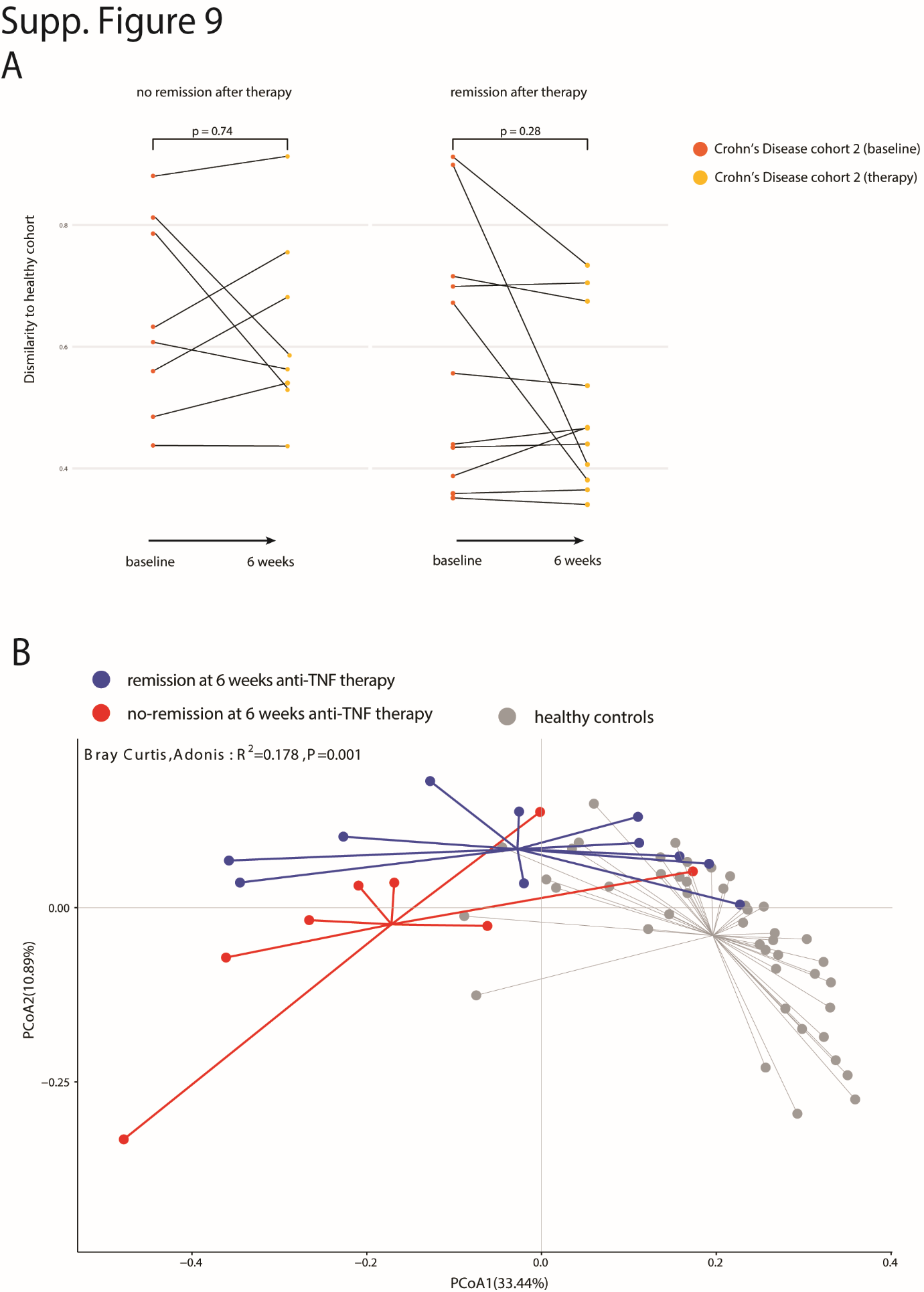
Supplemental Figure 9. The phenotypic signature of Crohn’s disease patients reaching remission following 6 weeks of anti-TNF therapy approaches that of healthy donors. (A)** The phenotypic microbiota signature differentiating Crohn’s disease patients from healthy controls (see Fig. 2) was used to assess the change in Bray-Curtis dissimilarity (β-diversity) of Crohn’s disease patients from baseline to 6 weeks of anti-TNF therapy relative to healthy donors. The Bray-Curtis dissimilarity of each sample of CD patient cohort 2, stratified into patients not reaching remission criteria and patients reaching remission after 6 weeks of therapy is plotted relative to the mean of the healthy donor cohort. **(B)** Principal Coordinate projection representing the Bray-Curtis dissimilarity between samples of remission and no-remission patients after 6 weeks of anti-TNF therapy in relation to healthy donors.

**
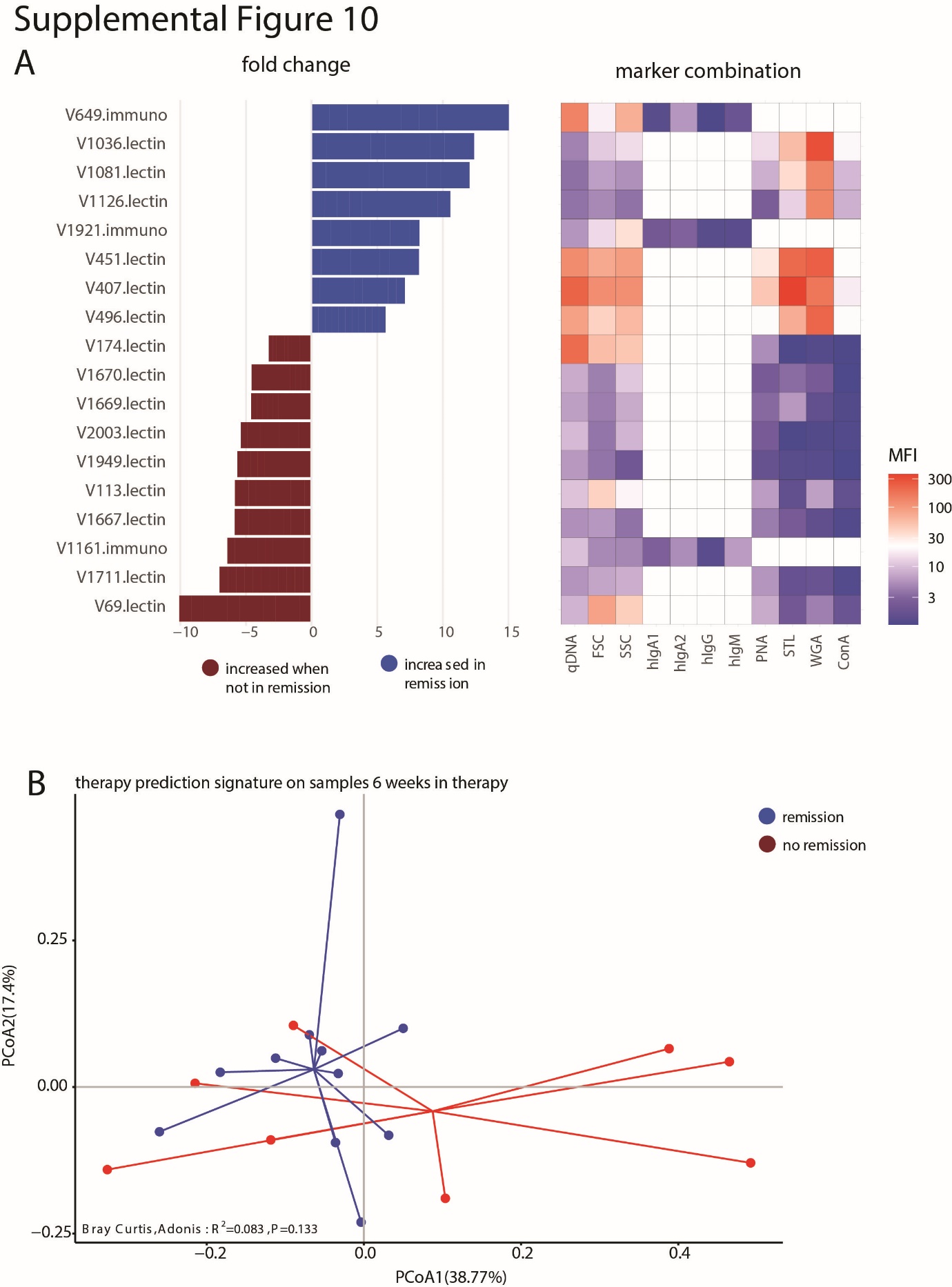
Supplemental Figure 10. The bacterial phenotypic signature predicting achievement of remission in Crohn’s disease patients with anti-TNF therapy at baseline**. **(A)** Representation of the total 18 clusters of the immunoglobulin and lectin panel selected by Wilcoxon test and recursive feature elimination differentiating between remission and no-remission CD patients of cohort 2 at baseline. Fold change indicates the difference in relative abundance of cells in that cluster between remission and no-remission CD patients. The heat map represents the phenotype of the cells defining that cluster as mean signal intensity normalized to the minimal and maximal signal observed in that parameter. **(B)** Principal Coordinate projection representing the Bray-Curtis dissimilarity between remission and no-remission patients after 6 weeks of anti-TNF therapy according to the 18 clusters selected to distinguish success of anti-TNF therapy at baseline.
